# Genomic insight into the integrative conjugative elements from ICE*Hpa1* family

**DOI:** 10.3389/fvets.2022.986824

**Published:** 2022-08-19

**Authors:** Huarun Sun, Junkai Zhang, Qingqing Miao, Yajun Zhai, Yushan Pan, Li Yuan, Fengbin Yan, Hua Wu, Gongzheng Hu

**Affiliations:** Department of Pharmacology and Toxicology, College of Veterinary Medicine, Henan Agricultural University, Zhengzhou, China

**Keywords:** multiresistance, *G. parasuis*, ICE, T4SS, ICE*Hpa1* family

## Abstract

Integrative conjugative elements (ICEs) are important carriers for disseminating resistance genes. We have previously reported a novel element ICE*Hpa1* carrying seven antibiotic resistance genes, which could be self-transmissible relying on the novel T4SS. To identify novel ICE*Hpa1* variants from 211 strains and novel T4SS encoded in ICE*Hpa1*, and to explore the relationships in these ICEs, four complete sequences of ICEs were identified by WGS analysis and antimicrobial susceptibility testing was determined by broth microdilution. In addition, a comparative analysis of these ICEs was conducted with bioinformatic tools, and the transfer abilities of these ICEs were confirmed by conjugation. Four ICE*Hpa1* variants ICE*Gpa1818*, ICE*Gpa1808*, ICE*Gpa1807*, and ICE*Gpa1815* with different resistance gene profiles were characterized, and their hosts showed different resistance spectrums. All ICEs shared the same backbone and were inserted into the tRNA^Leu^ site, and all resistance regions were inserted into the same target site between the accessory and integration regions. This study analyzed complete sequences of ICEs from the ICE*Hpa1* family and identified novel T4SS and insertion element IS*Gpa2*. Diverse resistance genes extensively exist in these ICEs, serving as a reservoir for resistance genes and facilitating their dissemination.

## Introduction

*Glaesserella parasuis* is a gram-negative bacterium usually involved in respiratory tract infections, polyarthritis, fibrinous polyserositis and meningitis in swine (1). *G. parasuis* is a primary swine pathogen causing respiratory disease worldwide that can result in huge economic losses (2). β-lactam, tetracycline and aminoglycoside antibiotics are the drugs of choice for treating infections in animals and zoonotic diseases in humans. Plasmid-mediated β-lactam, tetracycline and aminoglycoside resistance genes have been reported in *G. parasuis* (3–6), but these plasmid-mediated resistance genes are not self-transmissible through conjugation for lacking conjugal transfer proteins. The conventional view holds that prokaryotic evolution occurs due to clonal divergence and periodic selection (7). Horizontal gene transfer is another driving force in microbial evolution that allows microbes to acquire new genes and phenotypes, contributing to the diversification and adaptation of micro-organisms (8). Integrative conjugative elements (ICEs) are important carriers for horizontal gene transfer in bacterial populations (9). Hitherto, 28 ICEs families have been identified (https://bioinfo-mml.sjtu.edu.cn/ICEberg2/) in various Gram-positive and Gram-negative bacteria. We recently identified two ICEs from *G. parasuis* that carry multiple resistance genes, ICE*Hpa1* and its variant ICE*Gpa1804*, in the genomes from *G. parasuis* isolates (10). According to comparative sequence analysis, ICE*Hpa1* and ICE*Gpa1804* shared highly conserved ICE backbone, including replication, stabilization, type IV secretion system (T4SS) and integration without obvious structural homology with ICEs from reported families.

This study described four novel ICE*Hpa1* variants among 211 *G. parasuis* isolates that shared a common ICE backbone with ICE*Hpa1* and identified a novel T4SS. Moreover, we defined ICE*Hpa1* family and proposed a model for the emergence of ICE*Hpa1* elements that involve the continual recruitment of the different resistance gene modules. This work will shed light on the characteristics of the ICEs from ICE*Hpa1* family and puts forward innovative insights into the considerable diversity of genes and potential accessory functions encoded by the variable DNA in these ICEs.

## Materials and methods

### Bacterial strains and DNA sequence analysis

Two hundred and eleven strains were collected from pigs with respiratory diseases from pig farms and animal hospitals in Henan (*n* = 58), Hubei (*n* = 47), Hunan (*n* = 19), Anhui (*n* = 17), Jiangxi (*n* = 29), Shaanxi (*n* = 19), and Shanxi (*n* = 22) provinces of China from 2016 to 2019. The genomic DNA of the 211 strains was extracted using the QIAamp DNA Mini Kit (QIAGEN, Hilden, Germany) and then were sequenced *via* Illumina Hiseq platform and assembled by SPAdes (11). Resistance genes were determined *via* the CGE server (https://cge.cbs.dtu.dk/services/) (12). Subsequently, selected strains YHP1818, GHP1808, GHP1807 and YHP1815 with different antimicrobial resistance gene profiles were completely sequenced using a combined Illumina HiSeq and Nanopore sequencing approach. Initially, the quality of the raw sequence reads was checked using FastQC (Q30 > 85%). Adapter trimming of the Illumina reads was performed using Trimmomatic, and Nanopore reads shorter than 1,000 bp were removed. Sequencing reads as short-read and long-read data were assembled with Unicycler 0.4.4 with the hybrid assembly strategy (13, 14). Automated genome annotation was generated using the NCBI prokaryotic genome annotation pipeline (https://submit.ncbi.nlm.nih.gov/subs/genome/). Comparative analysis was conducted using the genome comparison visualizer Easyfig (15).

### Serotyping and MLST

Serovars of the host strains were determined using the primers that Jia mentioned (16). Seven housekeeping genes (*atpD, infB, mdh, rpoB, 6pgd, g3pd* and *frdB*) were amplified and sequenced as had been described (17), following registration of sequences at https://pubmlst.org/organisms/glaesserella-parasuis in terms of allele numbers and STs assignment. These data were analyzed *via* software available on the website.

### Susceptibility testing

Due to the unavailability of an approved method for *G. parasuis*, MICs of YHP1818, GHP1808, GHP1807 and YHP1815 were determined by the broth microdilution method (18) using cation-adjusted Mueller-Hinton broth containing 10% fetal bovine serum and 0.01% NAD, as suggested by a previous report (19). The tested antimicrobial agents were oxytetracycline, amoxicillin, enrofloxacin, streptomycin, gentamicin, tilmicosin, florfenicol, sulfamethoxazole/trimethoprim (19/1) and colistin. *Actinobacillus pleuropneumoniae* ATCC27090 and *Escherichia coli* ATCC 25922 were used as control strains.

### Evolutionary analyses of the core T4SS genes

Evolutionary analyses were implemented by MEGA7 (20), involving reference sequences derived from the NCBI database. Phylogenetic trees were created automatically by applying Neighbor-Join and BioNJ algorithms for core genes based on their respective amino acid sequences to further explore the evolution of the novel T4SS. The *traC*/*virB4, traD/virD4*, and *traI* genes encoded the typical T4SS proteins (21) and were ideal for analyzing the evolution of the T4SS.

### Conjugal transfer of the *G. Parasuis* ICEs

The circular intermediates of these ICEs were detected by PCR and sequencing with primers ICE-out-F and ICE-out-R as described previously (10). To investigate the conjugal transfer of these ICEs, this study adopted the YHP1818, GHP1808, GHP1807, and YHP1815 strains as the donors and *G. parasuis* V43 (rifampicin-resistance) as the recipient. As previously described (10), matings were performed with selection on TSA plates supplemented with 10% fetal bovine serum, 0.01% NAD, 8 mg/L oxytetracycline, and 100 mg/L rifampicin. Selected transconjugants were confirmed with PCR for the presence of the *virB4* gene using the primers, susceptibility testing and MLST.

### Nucleotide sequence accession numbers

The complete sequences of the four chromosomes carrying ICE*Gpa1818*, ICE*Gpa1808*, ICE*Gpa1807* and ICE*Gpa1815* have been submitted to GenBank with the following accession numbers: CP071487, CP071490, CP071491 and CP071489. The complete sequence of the plasmid p1807 in GHP1807 has been deposited in the GenBank database with accession number CP071492.

## Results

### General genetic features of host strains carrying ICEs and antimicrobial susceptibility

The whole-genome sequencing showed that 29 of 211 *G. parasuis* strains contain a conserved ICE*Hpa1*-like genetic backbone. Twenty nine strains were distributed into 12 different STs. The highest prevalence was observed for ST280 (6/29), followed by ST428 (5/29) and ST430 (5/29). Moreover, these 29 strains were assigned to 7 serovars. Serovars 4 (11/29) and 1 (10/29) were the most prevalent serovars. Table 1 shows the genetic features of 29 host strains carrying ICEs. Host strains YHP1818 [*tet*(B)], GHP1807 [*tet*(B), *strA, strB, aphA1, sul2*, and *floR*], GHP1808 [*tet*(B), *bla*_Rob−1_ and *aac(6)'-Ie-aph(2')-Ia*] and YHP1815 [*tet*(B), *bla*_Rob−1_, *strA, strB, aphA1*, and *sul2*] have different resistance gene profiles. The chromosomes of four host strains range from 2,289,620 bp to 2,435,338 bp and the GC contents of four chromosomes (40.03, 39.98, 40.02, and 39.93%) are very close. WGS analysis indicated that the strain GHP1807 contain one chromosome and a 5,215 bp plasmid, while YHP1818, GHP1808, and YHP1815 all contain just one chromosome. In the strain GHP1807, the *floR* gene was located on the small plasmid p1807, but other genes *tet*(B), *strA, strB, aphA1*, and *sul2* were located on the ICE*Hpa1* variant, i.e., ICE*Gpa1807*. In the other three strains, all resistance genes were located on the new ICEH*pa1* variants (ICE*Gpa1818*, ICE*Gpa1808*, and ICE*Gpa1815*), and all ICEs shared extensive sequence homology and highly conserved gene order. ICE*Hpa1* is the first to be identified and characterized (10) and thus was selected as a reference to define the ICE*Hpa1* family. The GC contents of the four ICEs are all lower than the rest of the genomes, and all ICEs are inserted into the tRNA^Leu^ sites. Table 2 shows the genetic features of the ICEs from ICE*Hpa1* family.

**Table 1:** General genetic features of 29 host strains carrying ICEs.

**Strain**	**Source**	**Year**	**Region**	**ST**	**Serovar**	**Resistance genes**
YHP1812	Trachea	2018	Henan	280	1	*bla*_Rob−1_, *tet*(B), *strA, strB, aphA1, sul2, erm*(Y)
YHP1813	Lung	2018	Henan	280	1	*bla*_Rob−1_, *tet*(B), *strA, strB, aphA1, sul2, floR*
YHP1806	Joint fluid	2018	Henan	280	1	*bla*_Rob−1_, *tet*(B), *strA, strB, aphA1, sul2, floR*
WHP1703	Joint fluid	2017	Anhui	280	1	*bla*_Rob−1_, *tet*(B), *strA, strB, aphA1, sul2, floR*
GHP1809	Trachea	2018	Jiangxi	280	1	*bla*_Rob−1_, *tet*(B), *strA, strB, aphA1, sul2, floR*
GHP1710	Lung	2017	Jiangxi	280	1	*bla*_Rob−1_, *tet*(B), *strA, strB, aphA1, sul2*
QHP1807	Lung	2018	Shaanxi	287	2	*bla*_Rob−1_, *tet*(B), *strA, strB, aphA1, sul2*
YHP1814	Lung	2018	Henan	287	1	*bla*_Rob−1_, *tet*(B), *strA, strB, aphA1, sul2*
**YHP1815**	**Lung**	**2018**	**Henan**	**287**	**2**	***bla*_Rob−1_, *tet*(B), *strA*, *strB*, *aphA1*, *sul2***
YHP1716	Lung	2017	Henan	470	10	*tet*(B), *strA, strB, aphA1, sul2*
YHP1801	Spleen	2018	Henan	160	10	*bla*_Rob−3_, *tet*(B), *strA, strB, aphA1, sul2, aac(6)'-Ie-aph(2')-Ia*
YHP170504	Lung	2017	Henan	288	8	*bla*_Rob−3_, *tet*(B), *strA, strB, aphA1, sul2, aac(6)'-Ie-aph(2')-Ia*
**GHP1807**	**Lung**	**2018**	**Jiangxi**	**429**	**11**	***tet*****(B)**, ***strA***, ***strB***, ***aphA1***, ***sul2***, ***floR***
EHP1711	Trachea	2017	Hubei	279	1	*bla*_Rob−1_, *tet*(B), *strA, strB, aphA1, catA3, aac(3)-IId, sul2*
EHP1804	Lung	2018	Hubei	279	1	*bla*_Rob−1_, *tet*(B), *strA, strB, aphA1, catA3, aac(3)-IId, sul2*
EHP1815	Lung	2018	Hubei	279	1	*bla*_Rob−1_, *tet*(B), *strA, strB, aphA1, catA3, aac(3)-IId, sul2*
SHP1606	Lung	2016	Shanxi	490	13	*tet*(B), *strA, strB, aphA1, sul2*
**YHP1818**	**Lung**	**2018**	**Henan**	**428**	**4**	***tet*** **(B)**
YHP1914	Lung	2019	Henan	428	4	*tet*(B)
XHP1802	Joint fluid	2018	Hunan	428	4	*tet*(B)
XHP1602	Joint fluid	2016	Hunan	428	4	*tet*(B)
EHP1718	Lung	2017	Hubei	428	4	*tet*(B)
EHP1802	Joint fluid	2018	Hubei	282	4	*tet*(B), *aadA1, cat*
YHP1825	Lung	2018	Henan	506	13	*bla*_Rob−3_, *tet*(B), *strA, strB, aphA1, sul2, aac(6)'-Ie-aph(2')-Ia*
**GHP1808**	**Lung**	**2018**	**Jiangxi**	**431**	**4**	***bla*_Rob−1_, *tet*(B), *aac(6)'-Ie-aph(2')-Ia***
GHP1811	Lung	2018	Jiangxi	431	4	*bla*_Rob−1_, *tet*(B), *aac(6)'-Ie-aph(2')-Ia*
SHP1708	Lung	2017	Shanxi	431	4	*bla*_Rob−1_, *tet*(B), *aac(6)'-Ie-aph(2')-Ia*
XHP1606	Lung	2016	Hunan	431	4	*bla*_Rob−1_, *tet*(B), *aac(6)'-Ie-aph(2')-Ia*
XHP1810	Lung	2018	Hunan	431	4	*bla*_Rob−1_, *tet*(B), *aac(6)'-Ie-aph(2')-Ia*

*The information of four host strains of ICEHpa1 variants were bolded*.

**Table 2:** General genetic features of ICEs from ICE*Hpa1* family and their host strains.

**ICE**	**Host**	**Size of chromosome** **(bp)**	**GC content of chromosome (%)**	**Size of ICE (bp)**	**GC content of ICE (%)**	**Site of insertion**	**Conjugation frequency**	**Reference or source**
Putative ICE in *Glaesserella sp*. 15-184	*Glaesserella sp*. 15-184	2,384,333	40.42	47,193	36.40	tRNA^Leu^	ND	29
**ICE** ***Gpa1818***	*G. parasuis* YHP1818	2,289,620	40.03	59,681	36.82	tRNA^Leu^	5.7 × 10^−5^	This study
**ICE** ***Gpa1808***	*G. parasuis* GHP1808	2,317,899	39.98	63,925	36.99	tRNA^Leu^	ND	This study
**ICE** ***Gpa1807***	*G. parasuis* GHP1807	2,435,338	40.02	68,454	38.27	tRNA^Leu^	6.4 × 10^−6^	This study
**ICE** ***Gpa1815***	*G. parasuis* YHP1815	2,357,562	39.93	68,581	37.86	tRNA^Leu^	3.3 × 10^−5^	This study
ICE*Hpa1*	*G. parasuis* YHP170504	2,520,015	39.64	68,922	37.42	tRNA^Leu^	6.1 × 10^−6^	10
ICE*Gpa1804*	*G. parasuis* EHP1804	2,398,603	39.97	71,880	38.84	tRNA^Leu^	4.3 × 10^−7^	CP069308
ICE*Asp1*	*Actinobacillus sp*. GY-402	2,458,209	46.00	72,978	37.71	tRNA^Leu^	5.8 × 10^−8^ or 4.3 × 10^−9^	30

*ND: not detectable. Sequence available through GenBank with the following accession numbers: Putative ICE in Glaesserella sp. 15-184 (CP023057), ICEGpa1818 (CP071487), ICEGpa1807 (CP071491), ICEGpa1808 (CP071490), ICEGpa1815 (CP071489), ICEHpa1 (CP054198), ICEGpa1804 (CP069308), and ICEAsp1 (CP062137). Four new ICEHpa1 variants were bolded*.

Strain YHP1818 exhibited high MIC of oxytetracycline (64 mg/L); GHP1808 showed high MICs of oxytetracycline (64 mg/L), amoxicillin (128 mg/L), streptomycin (128 mg/L), and gentamicin (128 mg/L); GHP1807 exhibited high MICs of oxytetracycline (64 mg/L), streptomycin (128 mg/L), sulfamethoxazole/trimethoprim (128 mg/L) and florfenicol (16 mg/L), and YHP1815 showed high MICs of oxytetracycline (64 mg/L), amoxicillin (16 mg/L), streptomycin (128 mg/L) and sulfamethoxazole/trimethoprim (128 mg/L) (Table 3).

**Table 3:** MICs and resistance genes in host isolates.

**Strain**	**Antimicrobial agents (mg/L)**													**Resistance genes**	**Reference or source**
	**OTC**	**TET**	**AMX**	**STR**	**KAN**	**GEN**	**ENF**	**CAP**	**FLO**	**CL**	**TIL**	**SF**	**SXT**		
15-184	-	-	-	-	-	-	-	-	-	-	-	-	-	None	29
YHP1818	64	-	2	2	-	1	1	-	<0.5	<0.5	0.5	-	4	*tet*(B)	This study
GHP1808	32	-	128	128	-	128	2	-	<0.5	<0.5	4	-	4	*tet*(B), *bla*_Rob−1_, *aac(6)'-Ie-aph(2')-Ia*	This study
GHP1807	64	-	2	128	-	1	4	-	16	<0.5	0.5	-	128	*tet*(B), *strA, strB, aphA1, sul2, floR*	This study
YHP1815	64	-	16	128	-	1	0.5	-	<0.5	<0.5	0.5	-	128	*tet*(B), *bla*_Rob−1_, *strA, strB, aphA1, sul2*	This study
YHP170504	64	-	64	128	-	256	8	-	<0.5	<0.5	1	-	≥512	*tet*(B), *bla*_Rob−3_, *aac(6)'-Ie-aph(2')-Ia, strA, strB, aphA1, sul2*	10
EHP1804	64	-	32	128	-	8	0.5	16	0.5	<0.5	0.5	-	128	*tet*(B), *bla*_Rob−1_, *strA, strB, aphA1, catA3, aac(3)-IId, sul2*	Cp069308
GY-402	-	>32	>128	-	>128	>256	-	>256	>32	4	>256	>512	-	*tet*(B), *bla*_Rob−3_, *aac(6)'-Ie-aph(2')-Ia, catT, strB, aphA1, sul2,erm*(T), *mcr-1, floR*	30

*“-”: not provided. OTC, oxytetracycline; TET, tetracycline; AMX, amoxicillin; STR, streptomycin; KAN, kanamycin; GEN, gentamicin; ENF, enrofloxacin; CAP, chloramphenicol; FLO, florfenicol; CL, colistin; TIL, tilmicosin; SF,sulfamethoxazole; SXT, sulfamethoxazole/trimethoprim*.

### Comparative sequence analysis of the T4SS

**Figure 1:**
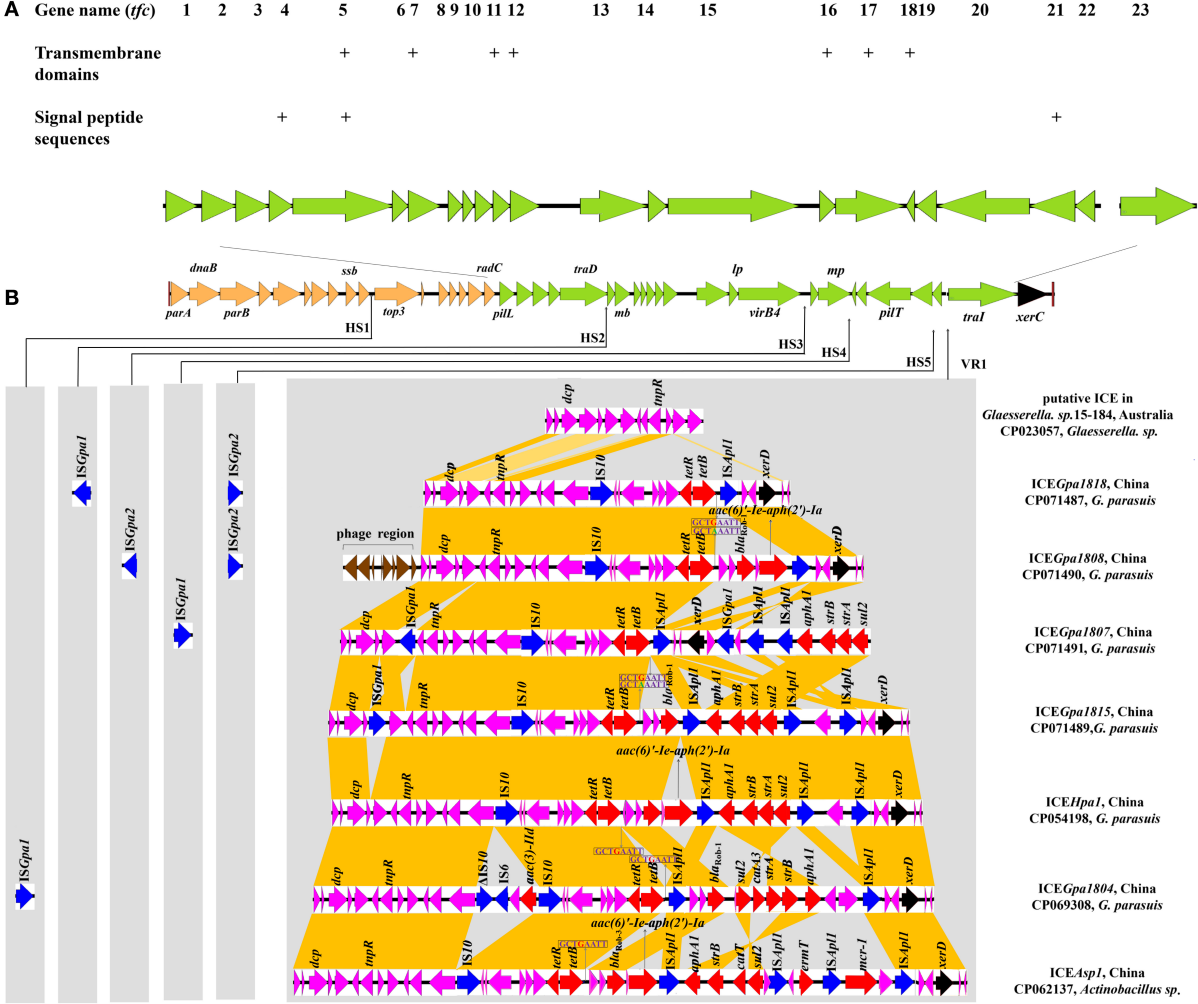
**(A)** Schematic representation of eight ICE*Hpa1* family ICEs and localization of the putative T4SS (green). This novel T4SS is encoded by 23 genes. Seven of the 23 ORFs from putative T4SS of the ICE*Hpa1* have transmembrane helices (indicated by plus signs) and 3 of the 23 genes are predicted to contain signal peptide sequences (indicated by plus signs). **(B)** Structural comparison between eight ICEs from ICE*Hpa1* family: the putative ICE in *Glaesserella sp*.15-184 (CP023057), ICE*Gpa1818* (CP071487), ICE*Gpa1807* (CP071491), ICE*Gpa1808* (CP071490), ICE*Gpa1815* (CP071489), ICE*Hpa1* (CP054198), ICE*Gpa1804* (CP069308), and ICE*Asp1* (CP062137). Thin arrows indicate the sites of insertion for hotspots (HS1-HS5) and variable region (VR1). The different genes are shown in different colors among these ICEs. The putative replication modules, T4SS modules, integration genes, phage proteins, accessory genes, resistance genes and IS elements are in orange, green, black, brown, purple, red, and blue, respectively. Regions of >68% homology in VR1 are marked by dark orange shading.

**Figure 2:**
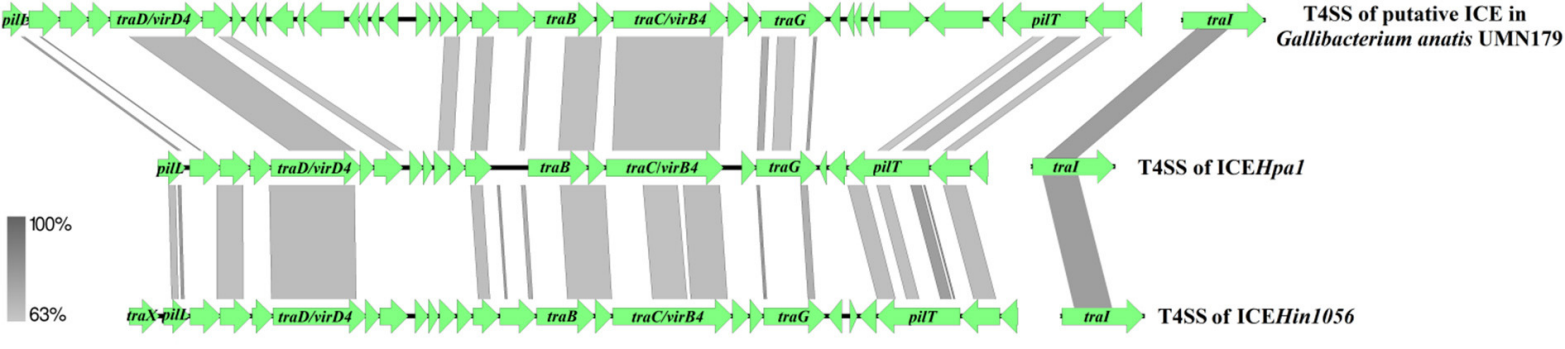
Compared the T4SS of ICE*Hpa1* (CP054198) with putative ICE in *Gallibacterium anatis* UMN179 (CP002667) and ICE*Hin1056* (AJ627386). Regions of >63% homology are marked by gray shading.

Comparative sequence analysis revealed that these four ICEs shared highly conserved ICE backbone, including replication, stabilization, T4SS and integration. Based on the bioinformatic analyses, 23 ORFs (*tfc1* to *tfc23*) that followed the putative replication module of ICE*Hpa1* were considered to encode a novel lineage T4SS (Figure 1A). Nineteen of 23 genes exhibited homology to known T4SS components (Table 4), a highly conserved module of the ICEs from the ICE*Hpa1* family, with 95 to 100% DNA similarity. T4SS of ICE*Hpa1* shares low sequence homology with ICE*Hin1056* (most closely, 14% cover and 72.21% identity) and putative ICE in *Gallibacterium anatis* UMN179 (14% cover and 71.79% identity) (Figure 2). Table 4 shows the deduced amino acid sequence similarities of the putative T4SS components of ICE*Hpa1* with the T4SS genes of ICE*Hin1056* and putative ICE in *Gallibacterium anatis* UMN179. T4SSs, with multi-subunit cell envelope spanning structures, comprise some genes encoding the Secretion channel, Pilus and Surface filamen (21, 22). Five ORFs were transcribed in a forward orientation, while the rest 18 ORFs were transcribed in the opposite direction (Figure 1A). Seven of the 23 ORFs from the putative T4SS cluster of the ICE*Hpa1* have transmembrane helices, playing an important role in transfer (Figure 1A). Furthermore, sequence analysis with SignalP 3.0 Server (23) revealed that 3 of the 23 genes of this gene cluster contain signal peptide sequences that are typical for genes involved in T4SSs.

**Table 4:** The deduced amino acid sequence similarities of the putative T4SS components of ICE*Hpa1* with the T4SS genes of ICE*Hin1056* and putative ICE in *Gallibacterium anatis* UMN179.

**T4SS components of ICE*Hpa1***	**T4SS components of putative ICE in *Gallibacterium anatis* UMN179**	**T4SS components of ICE*Hin1056***
Tfc1/PilL	93.33% cover, 40.48% identity	90.54% cover, 41.67% identity
Tfc2/Probable exported protein	93.65% cover, 46.19% identity	98.80% cover, 46.99% identity
Tfc3/Hypothetical protein	-	99.21% cover, 51.18% identity
Tfc4/Hypothetical protein	-	99.41% cover, 50.30% identity
Tfc5/VirD4/TraD	95.56% cover, 65.02% identity	99.72% cover, 64.73% identity
Tfc6/Hypothetical protein	-	74.51% cover, 30.26% identity
Tfc7/Membrane protein	99.57% cover, 48.93% identity	98.68% cover, 51.56% identity
Tfc8/Putative exported protein precursor	81.08% cover, 48.35% identity	85.34% cover, 45.45% identity
Tfc9/Hypothetical protein	-	-
Tfc10/Hypothetical protein	100.00% cover, 52.42% identity	-
Tfc11/Hypothetical protein	96.58% cover, 50.44% identity	95.12% cover, 40.17% identity
Tfc12/Putative exported protein precursor	91.86% cover, 60.59% identity	99.04% cover, 49.28% identity
Tfc13/TraB	99.59% cover, 46.82% identity	97.00% cover, 46.61% identity
Tfc14/Hypothetical protein	96.90% cover, 56.92% identity	95.45% cover, 56.82% identity
Tfc15/VirB4/TraC	99.13% cover, 65.40% identity	97.60% cover, 55.96% identity
Tfc16/Hypothetical protein	-	-
Tfc17/TraG	95.63% cover, 45.24% identity	97.38% cover, 42.77% identity
Tfc18/Hypothetical protein	-	-
Tfc19/Hypothetical protein	-	85.19% cover, 31.30% identity
Tfc20/PilT	74.20% cover, 52.67% identity	99.40% cover, 52.62% identity
Tfc21/Hypothetical protein	97.75% cover, 55.26% identity	94.48% cover, 58.78% identity
Tfc22/Hypothetical protein	100.00% cover, 37.32% identity	92.41% cover, 40.15% identity
Tfc23/TraI	99.38 cover, 45.54% identity	96.85% cover, 51.54% identity

The bioinformatic analysis showed *tfc5* exhibits homology with *traD/virD4*, a gene encoding T4SS conjugal transfer protein VirD4 in the DNA transport (24). *tfc15* is homologous to *traC*/*virB4* gene encoding an important T4SS component VirB4, which is required for the assembly of the system and substrate transfer (25). *tfc13* and *tfc17* are homologous to *traB* and *traG*, respectively, whose products are required for type IV secretion pilus assembly. Moreover, *tfc23* has homology to *traI* gene encoding a relaxase to transfer N-terminally-fused Cre to target cells (26). *tfc1* and *tfc20* are homologous with *pilL* and *pilT*, respectively, and encode outer membrane lipoproteins responsible for thin pilus biosynthesis (27, 28).

### Evolutionary analyses of the TraC/VirB4, TraD/VirD4 and trai proteins

**Figure 3:**
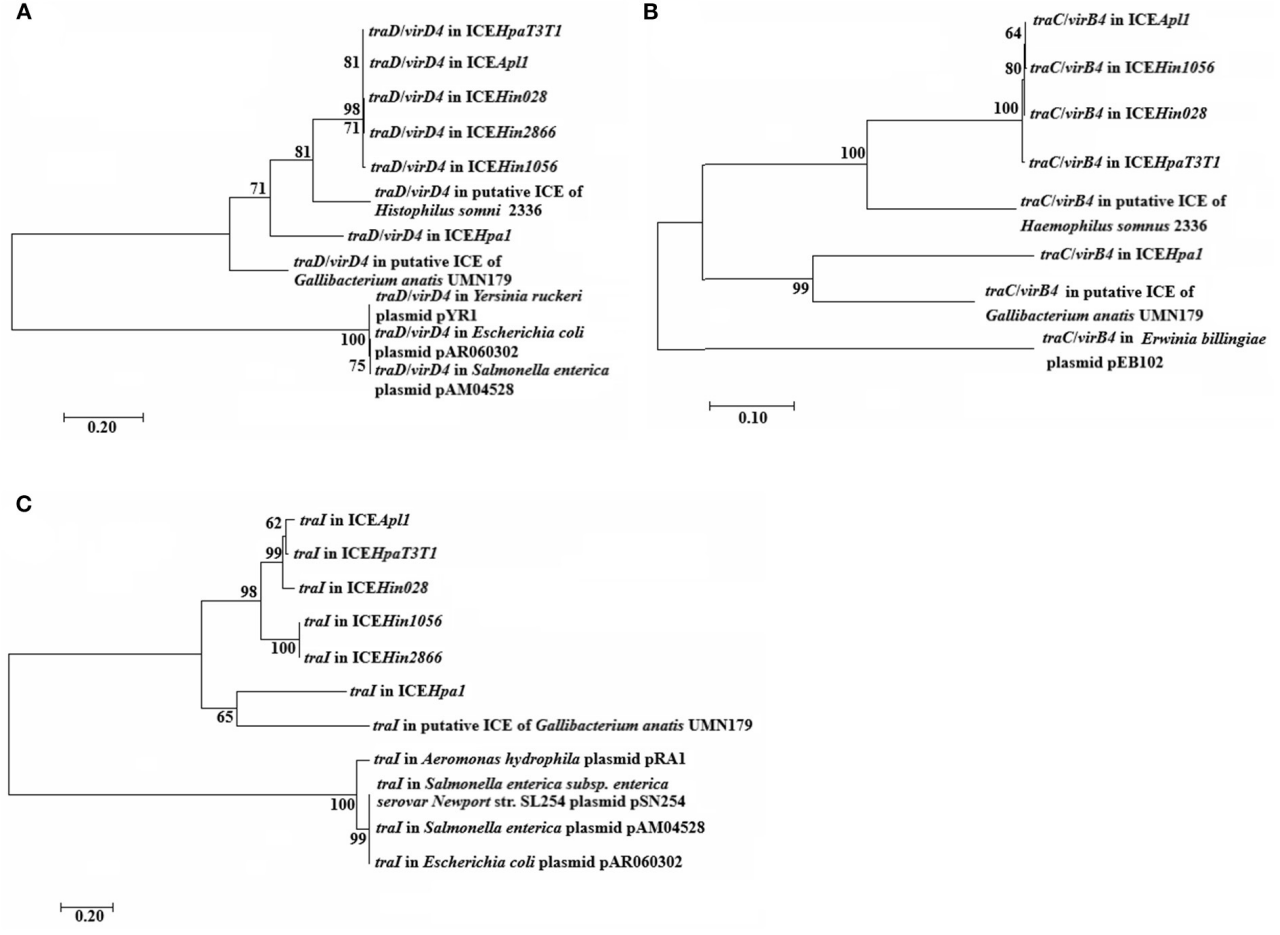
Phylogenetic analysis of **(A)** *traD*/*virD4*, **(B)** *traC*/*virB4*, and **(C)** *traI* genes. Amino acide sequences of the indicated these core genes were used to generate the phylogenetic trees shown. Bootstrap values are indicated at branch points. The individual scale bars represent genetic distances and reflect the number of substitutions per residue.

The phylogenetic tree analysis indicate that the TraC/VirB4, TraD*/*VirD4 and TraI proteins are on a separate branch of the phylogenetic tree, respectively (Figure 3). Moreover, the trees for TraC/VirB4 and TraI proteins are extremely similar, of which the ICE*Hpa1* and putative ICE in *Gallibacterium anatis* UMN179 formed an evolutionarily separate group that differed from other ICEs and plasmids. Besides, TraD*/*VirD4 in ICE*Hpa1* exhibits a different branching pattern alongside ICE*Hin1056* members, putative ICE in *Haemophilus somnus* 2,336 and putative ICE in *Gallibacterium anatis* UMN179. From the silico bioinformatic analysis, it could be concluded that the gene cluster encoded structural components of a novel putative T4SS, which is evolutionarily different from previously described systems.

### Analysis of VR1 regions in ICE*Hpa1* family ICEs

Apart from the core genes, five potential “hotspots” (HS1-HS5) and one “variable region”(VR1) were interspaced into the conserved backbone of ICEs. Two novel insertion elements, IS*Gpa1* and IS*Gpa2*, were distributed between five hotspots (HS1-HS5) and the variable region (VR1). IS*Gpa1* and IS*Gpa2* with high host specificity, however, were only present in the ICEs from *G. parasuis*.

This study coded variable DNAs in VR1 for resistance to antibiotics and bacteriophage infection, which conferred adaptive functions to various environments. The VR1 mainly consisted of three segments in an accessory genes region, a multidrug-resistant region (MRR) and an integration region. Unlike other ICEs, upstream of three segments, a 5,137 bp cassette encoding eight proteins, was predicted as a prophage-related element in ICE*Gpa1808*. The contents of the accessory genes region and the integration region in other resistance ICEs were almost identical except for ICE*Gpa1807* and ICE*Gpa1815*. The integration region of VR1 in ICE*Gpa1807* was inserted into the MRR, which varied in the position and direction from other resistance ICEs in Figure 1B. An insertion element, IS*Gpa1*, was found downstream of the zincin-like metallopeptidase domain-containing protein. Similarly, the IS*Gpa1* was also observed in the accessory genes region of ICE*Gpa1815*, with a different direction from ICE*Gpa1807*.

Compared with the putative ICE in the genome of *Glaesserella sp*.15-184 (28), except for ICE*Gpa1807*, MRRs were inserted into the same site in the resistant ICEs between the segment *tnpR*-*hp* and integration gene *xerD*. The MRR of ICE*Gpa1818* was characterized by a truncated transposon Tn*10*, remaining two regulatory genes *lysR* and *arsR*, a sodium/glutamate symporter, an antibiotic biosynthesis monooxygenase, an amino acid-binding protein, a hypothetical protein, the repressor gene *tetR*, and the tetracycline resistance gene *tet*(B), as well as 83 bp of the downstream region. Correspondingly, the host strain harboring ICE*Gpa1818* showed a high MIC of oxytetracycline (64 mg/L). The truncated Tn*10* was also found on the left of the MRR of ICE*Gpa1808*. Moreover, a 2,804 bp segment carrying genes *bla*_Rob−1_ and *aac(6)'-Ie-aph(2')-Ia* from *G. parasuis* pQY431 was observed immediately downstream of the truncated transposon Tn*10*. Correspondingly, the host strain harboring ICE*Gpa1808* showed high MICs of oxytetracycline (32 mg/L), amoxicillin (128 mg/L), streptomycin (128 mg/L) and gentamicin (128 mg/L). The resistance gene region of ICE*Gpa1807* comprised two parts with five different resistance genes and four complete insertion sequences (one IS*10* copy and three IS*Apl1* copies). A truncated Tn*10* and an IS*Apl1* were identified upstream of the integration region of VR1. Downstream of the integration region of VR1, two IS*Apl1* copies, the four resistance genes *aphA1, strB, strA* and *sul2*, were detected and all oriented in the same direction. The host strain harboring ICE*Gpa1807* showed high MICs of oxytetracycline (64 mg/L), streptomycin (128 mg/L), and sulfamethoxazole/trimethoprim (128 mg/L) accordingly. Immediately downstream of the truncated Tn*10*, a *bla*_Rob−1_, an intact transposon Tn*6742*, and an additional IS*Apl1* element were found in the MRR of ICE*Gpa1815*. The host strain harboring ICE*Gpa1815* showed high MICs of oxytetracycline (64 mg/L), amoxicillin (16 mg/L), streptomycin (128 mg/L) and sulfamethoxazole/trimethoprim (128 mg/L).

**Figure 4:**
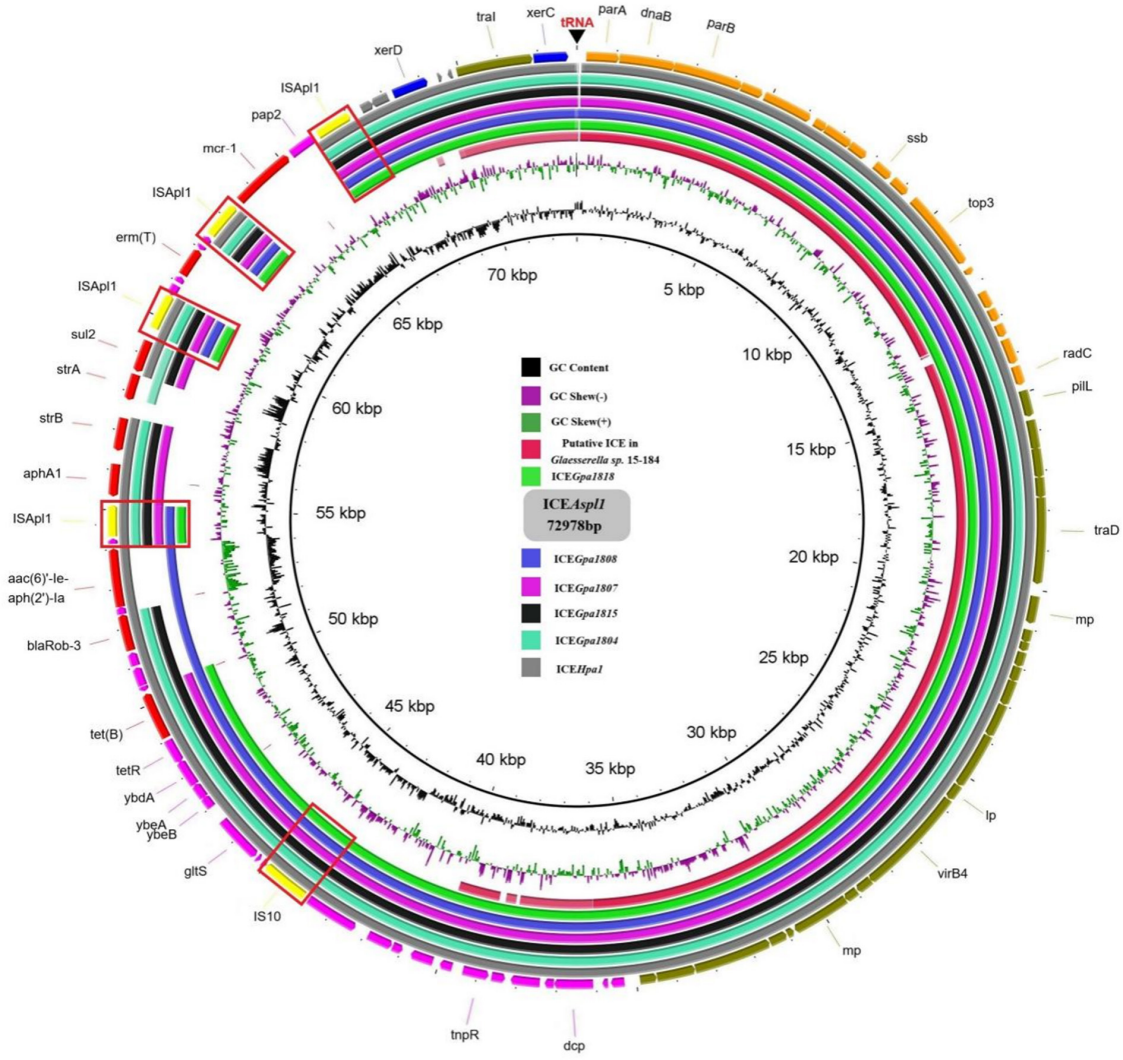
Comparison of the circular form of the ICE*Hpa1* family ICEs. The outer ring comprises the coding sequences (CDSs) of the ICE*Asp1*. Circular form of the ICE*Asp1* was used as a reference to compare with the the putative ICE in *Glaesserella sp*.15-184 (CP023057), ICE*Gpa1818* (CP071487), ICE*Gpa1808* (CP071490), ICE*Gpa1807* (CP071491), ICE*Gpa1815* (CP071489), ICE*Gpa1804* (CP069308), and ICE*Hpa1* (CP054198). Key features of the ICE*Asp1* are highlighted in different colors, replication genes and stability-associated genes are in orange, transfer-associated genes are in olive, IS elements are in yellow, ARGs are in red, other genes are in purple and integrase genes are in blue. IS elements were marked by the boxes.

**Figure 5:**
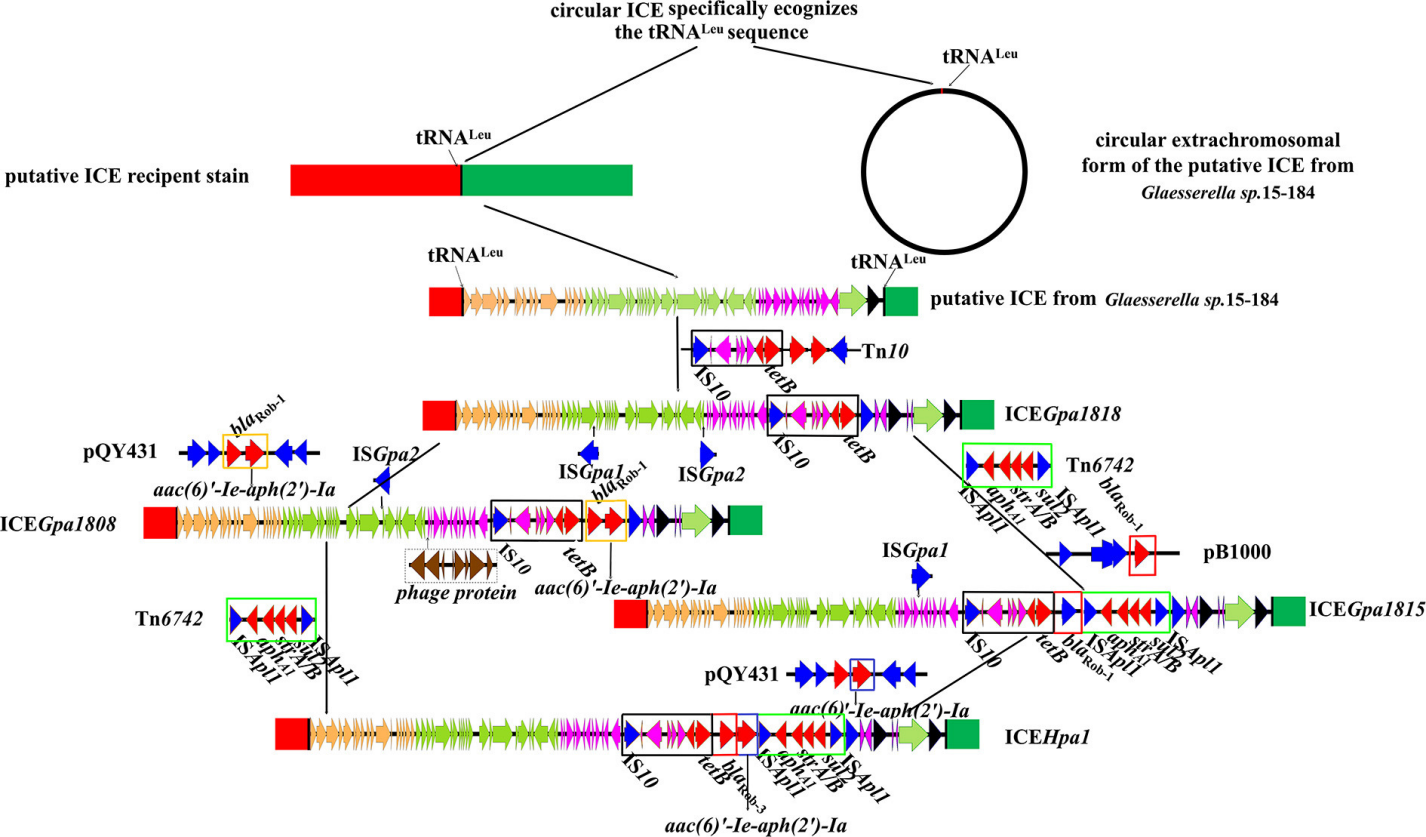
Schematic representation of the stepwise evolution process of ICE*Hpa1*. Genes are presented as broad arrows, with the arrowhead indicating the direction of transcription. The reference sequences include the putative ICE in *Glaesserella sp*.15-184 (CP023057), ICE*Gpa1818* (CP071487), ICE*Gpa1808* (CP071490), ICE*Gpa1815* (CP071489), ICE*Hpa1* (CP054198), transposon Tn*10* (AF162223), transposon Tn*6742* (MN844034), plasmid pQY431 (KC405065), and plasmid pB1000 (DQ840517).

Apart from ICEs from *G. parasuis*, two additional putative ICEs from *Glaesserella sp*. (29) and *Actinobacillus sp*. (30) from Pasteurellaceae were observed to contain similar genetic backbone. According to comparative analysis of MRRs from ICE*Hpa1* family ICEs, antibiotic resistance genes *tet*(B), *bla*_Rob−1_, *bla*_Rob−3_, *aac(6)'-Ie-aph(2')-Ia, aphA1, strA, strB, sul2, aac(3)-IId, catA3, erm*(T) and *mcr-1* were observed in the MRRs of ICE*Hpa1* family ICEs (Figure 4). Truncated Tn*10* harboring the antibiotic resistance determinant *tet*(B) existed in all resistance ICEs without *tetC, tetD* and IS*10*-R. The truncated Tn*10* in tested ICEs, however, did not abolish the tetracyclines resistance activity of the host strain. An 8 bp' GCTGAATT' or 'GCTAAATT' was found immediately downstream of the 6,687 bp truncated Tn*10* in ICEs of this family (Figure 1B), indicating that the specific locus was recognized, and the Tn*10* in these ICEs was truncated. Based on the acquired truncated Tn10, other acquired resistance genes [*bla*_Rob−1_, *bla*_Rob−3_, *aac(6)'-Ie-aph(2')-Ia*] were probably accumulated from small plasmids by homologous recombination, but *aphA1, strA, strB, sul2, catA3, erm*(T) and *mcr-1* were acquired by a series of insertions that were meditated by IS*Apl1* (Figures 4, 5). The tandem multiplication of the IS*Apl1*-flanked antibiotic resistance genes probably caused the expansion of the MRRs.

### Two potential formation processes of MRR of ICE*Hpa1*

In most cases, all ICEs exhibited extensive DNA sequence homologies and preserved gene order, but the main difference lay in their MRRs. High sequence similarities (some of 100%) were observed, especially in the putative replication and T4SS modules (Figures 4, 5). Based on the bioinformatic analyses of a series of ICEs of the ICE*Hpa1* family, we deduced two possible formation processes of MRR of ICE*Hpa1* (Figure 5).

Primitively, ICE*Hpa1*-like circular extrachromosomal form with no resistance gene recognized specific tRNA^Leu^ site and then was integrated into the potential host chromosome. The putative ICE in the genome of *Glaesserella sp*.15-184 was regarded as the original structure of the ICE*Hpa1*. ICE*Gpa1818* was formed as the putative ICE in the genome of *Glaesserella sp*.15-184 captured partial transposon Tn*10* DNA sequences. Subsequently, episomal small transposon Tn*6742* was inserted downstream of the *tet* (B) of ICE*Gpa1818*, thus forming a new element. When the new element and small plasmid pB1000 collided under certain conditions, this element captured the *bla*_Rob−1_ gene *via* homologous recombination to form a novel element ICE*Gpa1815*. Ultimately, ICE*Gpa1815* got the bifunctional enzyme AAC(6')-Ie-APH(2")-Ia from small plasmid pQY43 *via* homologous recombination.

The other potential formation process of MRR of ICE*Hpa1* was observed *via* comparative sequence analysis. The putative ICE in the genome of *Glaesserella sp*.15-184 captured partial transposon Tn*10* DNA sequences, and acquired the segment carrying the genes *bla*_Rob−1_ and *aac(6)'-Ie-aph(2')-Ia via* homologous recombination from *G. parasuis* pQY43. Subsequently, the intact transposon Tn*6742* was acquired *via* IS*Apl1*-mediated insertion.

### Conjugal transfer of four ICEs

Inverse PCR with primers were positive for four host strains carrying ICEs which indicated the presence of the intermediate circular form of ICE*Gpa1818*, ICE*Gpa1807*, ICEGpa1815, and ICE*Gpa1808*. Transfers occurred with frequencies of 5.7 × 10^−5^, 6.4 × 10^−6^ and 3.3 × 10^−5^ transconjugants per donor cell (YHP1818, GHP1807, and YHP1815) for strain V43, respectively. However, conjugation experiments failed to obtain transconjugants of GHP1808, despite repeated attempts. Together, these results demonstrated that the ICE*Gpa1818*, ICE*Gpa1807*, and ICE*Gpa1815* were active and transferable ICE elements. The conjugation frequencies of the ICEs tested were recorded in Table 2.

### Identification of a novel insertion element, IS*Gpa2*

A novel DNA segment with a structural characteristic of insertion elements was identified in these novel ICEs with the BLAST analysis, and designated as IS*Gpa2* according to ISs nomenclature (https://www-is.biotoul.fr/). The nucleotide sequence of insertion sequence IS*Gpa2* was deposited in the ISFinder database.

IS*Gpa2* was 1,180-bp long with a GC content of 42.29%, containing a single 972 bp ORF. Comparisons of the sequence with the ISFinder and GenBank databases revealed that IS*Gpa2* belonged to the IS*110* family. The IS*Gpa2* transposase exhibited extensive amino acid sequence homology with the putative transposase of IS*Nme5* from *Neisseria meningitidis* FAM18 (69.4% coverage with 56.6% identity) and IS*Pye59* from *Paracoccus yeei* TT13 (50.5% coverage with 33.0% identity). Like other members of the IS*110* family, no DRs or IRs were found in IS*Gpa2*. IS*Gpa2* also exists in the *G. parasuis* reference strains SH0104 (CP024412), SH0165 (CP001321), HPS412 (CP041334) and some other isolates.

### Analysis of the *FloR*-carrying plasmid in GHP1807

**Figure 6:**
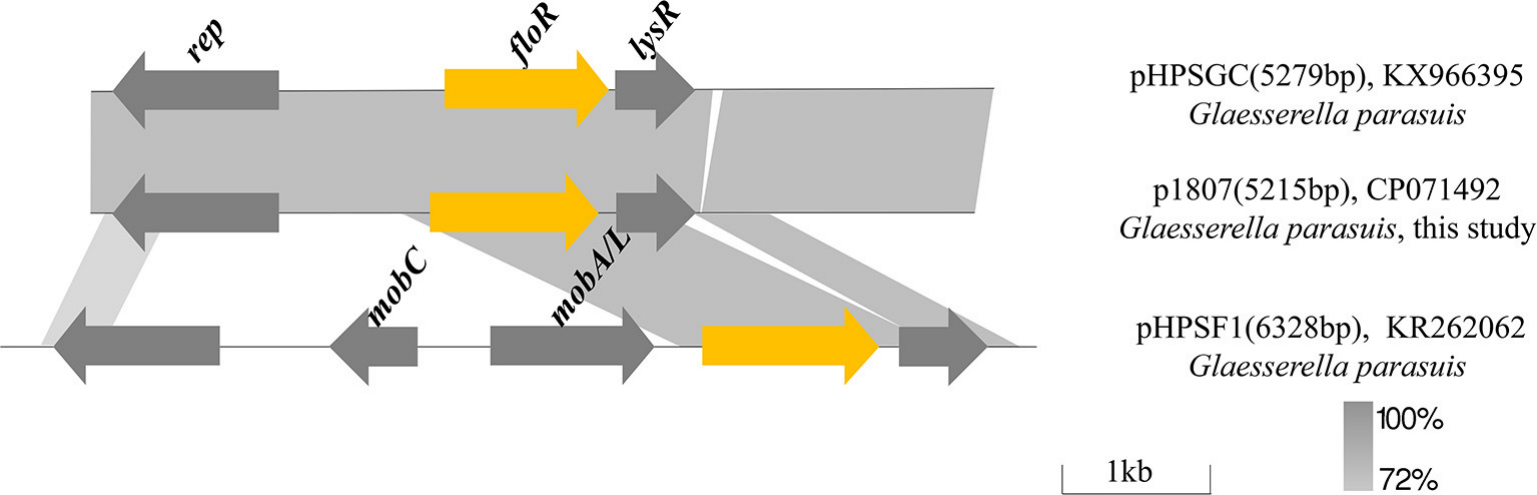
Comparison of plasmids p1807 (CP071492), pHPSGC (KX966395), and pHPSF1 (KR262062). Arrows indicate gene positions and transcriptional orientation. Regions of >72% homology are marked by gray shading.

In GHP1807, plasmid p1807 is 5,215 bp and consists of 3 ORFs encoding the florfenicol resistance protein FloR, transcriptional regulator LysR and a potential Rep protein involved in plasmid replication (Figure 6). Comparative sequence analysis revealed that p1807 almost shares 100% identity with the pHPSGC but misses 64 bp. The segment carrying *floR* and *lysR* also exists in pHPSF1 with different backbones from *G. parasuis*. In pHPSF1, the segment carrying *floR* and *lysR* was integrated to the plasmid backbone, which consists of mobilization genes *mobA/L* and *mobC*, and *rep* gene. It seems that the segment carrying *floR* and *lysR* originating from pHPSF1 was integrated to new plasmid backbone to generate a novel plasmid p1807.

## Discussion

According to previous reports, plasmids are the main carriers to disseminate resistance genes among *G. parasuis* strains (3–6). In this study, 29 strains contain the conserved ICE genetic backbone among the 211 *G. parasuis* strains, indicating that ICEs are the predominant carriers for various resistance genes in *G. parasuis*. Strains YHP1818, GHP1807, GHP1808, YHP1815, YHP170504, and EHP1804 contained similar ICEs (ICE*Gpa1818*, ICE*Gpa1808*, ICE*Gpa1807*, ICE*Gpa1815*, ICE*Hpa1*, and ICE*Gpa1804*, respectively) that were isolated from various places (Henan, Jiangxi, Jiangxi, Henan, Henan and Hubei). Similar situations have been observed in the other species of bacteria. Strains contained highly similar genetic backbones but were isolated at divergent places and in different years (31, 32).

Host strains carrying ICEs were distributed into 7 different serovars (serovar 4, 1, 2, 10, 13, 8 and 11), which suggested a tendency for these ICEs from the ICE*Hpa1* family to be inherited by horizontal transfer rather than vertical transmission. In addition, these ICEs tended to have greater mobility among *G. parasuis* isolates. Meanwhile, 21 *G. parasuis* host strains were distributed with two serovars (serovar 4 and 1), indicating that these serovars clinical isolates were the main host strains for ICEs, and no restriction barriers hindered the transfer between these serovars. However, multiple conjugation experiments for GHP1808 failed, the reason for which is unknown. Non-transferability of ICE*Gpa1808* may be caused by the the insertion of the prophage-related element upstream the MRR or the transfer frequency in this strain is rather low.

ICEs from ICE*Hpa1* family are typically mosaic and modular and grouped on the element as they contained functional modules from different sources. None of the ICEs from ICE*Hpa1* family contained nucleotide sequences, indicating recent acquisition from other families than Pasteurellaceae. This observation proved that the ICE*Hpa1* family was evolving by descent within its host species without recombining with other species-specific families. Additionally, this ICE*Hpa1* family appeared to be shared only by strains associated with swine. The characteristics of *traC*/*virB4, traD/virD4* and *traI* trees revealed that the great degree of recombination covered these core genes common evolutionary history or/and that these genes had evolved independently. Furthermore, different core genes co-existed in a host chromosome in tandem *via* recombination to form a novel T4SS.

ICEs are integrated into and excises from DNA *via* an ICE-encoded recombinase (9). Putative ICE in the genome of *Glaesserella sp*.15-184 carrying recombinase XerC was proved to integrate into, and excise from the host chromosome (29), and such was the case of ICE*Hpa1* harboring recombinase XerC and XerD. These findings suggested that ICE*Hpa1* family ICEs were probably integrated into and excised using an ICE-encoded XerC recombinase, while resistance regions were integrated at the specific attachment site by XerD recombinase. A range of resistance genes was located in the genome's ICEs (Table 3, Figure 1B), contributing to bacteria's diversification and adaptation. The resistance regions of these ICEs were likely assembled with different parts from other small plasmids from *G. parasuis*, and other transposons. These resistance genes conferred resistance to many antibacterial agents, including β-lactams, tetracyclines, aminoglycosides, sulfonamides, chloramphenicol, and macrolides colistin. In each case, the resistance genes are always carried on small transposons, with tetracycline resistance genes on Tn*10*, β-lactamase-encoding genes on Tn*3*, colistin resistance genes on Tn*6330* and aminoglycoside and sulfonamide resistance genes on Tn*6742* (10, 30). Tn*10* was one of the most extensively studied transposable elements and an original case of small composite transposon where two same IS*10* elements cooperated to mediate the transposition of antibiotic resistance genes (33). Consisting of indirectly repeating insertion sequences IS*10*-L and IS*10*-R flanking *tet* genes, Tn*10* was involved in tetracyclines resistance (34, 35). Truncated Tn*10* carrying the antibiotic resistance determinant *tet* (B) presented in all resistance ICEs that did not contain the *tetC, tetD* and IS*10*-R. Truncated Tn*10* in tested ICEs, however, did not abolish the tetracyclines resistance activity of the host strain, which verified Richard's previous conclusion that the genes *tetC* and *tetD* were not involved in tetracycline resistance in the transposon Tn*10* (34, 35). Most transposons contained drug-resistant cassettes in divergent ICEs, which increased the possibility for these resistance genes to be captured after acquiring ICEs by host strains. Furthermore, unlike other ICEs from the ICE*Hpa1* family, upstream of three segments and eight phage-related ORFs were found in ICE*Gpa1808*, but the role of the phage cassette in the resistance ICEs transmission remained unclear.

A recent study confirmed that ICE*Asp1* from *Actinobacillus sp*. is a new member of the ICE*Hpa1* family (30). ICE*Asp1* is the first resistance ICE related to the ICE*Hpa1* family other than *G. parasuis*. Compared with the resistance gene region of ICE*Hpa1*, the *strA* gene was replaced by a novel chloramphenicol resistance gene *catT* in the resistance gene region of ICE*Asp1*. An *erm* (T) gene and a putative transposon Tn*6330* carrying colistin resistance gene *mcr-1* were located immediately downstream of these modules. Colistin has been viewed as the last resort in treating Gram-negative bacterial infections (36). The emergence of ICE*Asp1* carrying a colistin resistance gene *mcr-1* signified that the ICEs from ICE*Hpa1* family could act as a reservoir for *mcr-1* and accelerate the spread of colistin resistance.

Two potential formation processes of MRR of ICE*Hpa1* were deduced from the dataset, suggesting that the spread of ICE*Hpa1*-mediated drug resistance was not strictly related to specific evolution processes. Various resistance genes were gradually captured by ICE*Hpa1*. Conceivably, the ICEs from the ICE*Hpa1* family as the carriers should have a larger resistance gene load than small plasmids derived from Pasteurella (3–6). Despite limited knowledge of resistance genes-mobilizing vehicles, it could be deduced that resistance genes embedded in ICE*Hpa1* structures were capable of broader horizontal transmission with the benefit of abundant is elements.

The structural comparison showed that the formation of ICE*Hpa1* probably resulted from the abundance of IS*Apl1*, IS*Gpa1* and IS*Gpa2* elements. Interestingly, IS*Apl1*, originally identified in the *A. pleuropneumoniae* (37), was observed in the resistance gene region among all resistance ICEs from the ICE*Hpa1* family. IS*Apl1* promoted the adaptive evolution of bacteria and helped the host adapt to distinct environments by accumulating resistance genes. Multiple resistance genes *aphA1, strB, catT, sul2, erm* (T) and *mcr-1* were observed in ICE*Asp1*, involving a series of insertion events meditated by IS*Apl1*. Moreover, two newly identified IS elements with high host specificity, IS*Gpa1* and IS*Gpa2*, only exist in the *G. parasuis* reference strains. Except for ICE*Hpa1*, IS*Gpa1* and IS*Gpa2* were sporadically inserted in potential “hotspots” and “variable region” of the other five resistance ICEs from *G. parasuis*. Multiple copies IS*Gpa1* and IS*Gpa2* could serve as crossover points for homologous recombination events and play a vital role in genome flexibility, adaptation, and evolution of *G. parasuis* genomes. Moreover, the two novel insertion elements are critical to transferring ICEs among *G. parasuis* strains.

## Conclusions

This study revealed that the diverse ICEs from the ICE*Hpa1* family recently emerged in the isolates of *G. parasuis, Glaesserella sp*. and *Actinobacillus sp*. from Pasteurellaceae. A novel T4SS from these ICEs was characterized, and potential “hotspots” (HS1-HS5) and one “variable region” (VR1) were defined in this study. In addition, the potential formation processes of MRR of ICE*Hpa1* were deduced, and IS*Gpa2*, a novel insertion element, was identified. The ICEs from the ICE*Hpa1* family are the predominant carriers for various resistance genes. More in-depth research on ICE*Hpa1* family ICEs will be carried out in our laboratory.

## Data availability statement

The datasets presented in this study can be found in online repositories. The names of the repository/repositories and accession number(s) can be found in the article/supplementary material.

## Author contributions

GH and HS conceived and designed the experiments. HS, JZ, QM, FY, and HW produced the data. HS, YZ, YP, LY, and HW analyzed the data. HS, HW, and GH wrote the paper. All authors contributed to the article and approved the submitted version.

## Funding

This study was financed by the National Key Research and Development Program of China [2016YFD05101304].

## Conflict of interest

The authors declare that the research was conducted in the absence of any commercial or financial relationships that could be construed as a potential conflict of interest.

## Publisher's note

All claims expressed in this article are solely those of the authors and do not necessarily represent those of their affiliated organizations, or those of the publisher, the editors and the reviewers. Any product that may be evaluated in this article, or claim that may be made by its manufacturer, is not guaranteed or endorsed by the publisher.
